# Screen time and mental health: a prospective analysis of the Adolescent Brain Cognitive Development (ABCD) Study

**DOI:** 10.1186/s12889-024-20102-x

**Published:** 2024-10-07

**Authors:** Jason M. Nagata, Abubakr A.A. Al-Shoaibi, Alicia W. Leong, Gabriel Zamora, Alexander Testa, Kyle T. Ganson, Fiona C. Baker

**Affiliations:** 1 Department of Pediatrics, University of California, San Francisco, 550 16th Street, 4th Floor, Box 0503, San Francisco, CA 94143 USA; 2 Icahn School of Medicine at Mount Sinai, 1 Gustave L. Levy Pl, New York, NY 10029 USA; 3 Department of Management, Policy and Community Health, University of Texas Health Science Center at Houston, 7000 Fannin St, Houston, TX 77030 USA; 4 Factor-Inwentash Faculty of Social Work, University of Toronto, 246 Bloor St W, Toronto, ON M5S 1V4 Canada; 5 Center for Health Sciences, SRI International, 333 Ravenswood Ave, Menlo Park, CA 94025 USA; 6 School of Physiology, University of the Witwatersrand, 1 Jan Smuts Avenue, Braamfontein, Johannesburg, 2000 South Africa

**Keywords:** Screen time, Adolescents, Depression, Anxiety, Oppositional defiant disorder, Conduct disorder, ADHD, Somatic, Social media, Video games, Television, Digital technology, Digital media

## Abstract

**Background:**

Despite the ubiquity of adolescent screen use, there are limited longitudinal studies that examine the prospective relationships between screen time and child behavioral problems in a large, diverse nationwide sample of adolescents in the United States, which was the objective of the current study.

**Methods:**

We analyzed cohort data of 9,538 adolescents (9–10 years at baseline in 2016–2018) with two years of follow-up from the Adolescent Brain Cognitive Development (ABCD) Study. We used mixed-effects models to analyze associations between baseline self-reported screen time and parent-reported mental health symptoms using the Child Behavior Checklist, with random effects adjusted for age, sex, race/ethnicity, household income, parent education, and study site. We tested for effect modification by sex and race/ethnicity.

**Results:**

The sample was 48.8% female and racially/ethnically diverse (47.6% racial/ethnic minority). Higher total screen time was associated with all mental health symptoms in adjusted models, and the association was strongest for depressive (B = 0.10, 95% CI 0.06, 0.13, *p* < 0.001), conduct (B = 0.07, 95% CI 0.03, 0.10, *p* < 0.001), somatic (B = 0.06, 95% CI 0.01, 0.11, *p* = 0.026), and attention-deficit/hyperactivity symptoms (B = 0.06, 95% CI 0.01, 0.10, *p* = 0.013). The specific screen types with the greatest associations with depressive symptoms included video chat, texting, videos, and video games. The association between screen time and depressive, attention-deficit/hyperactivity, and oppositional defiant symptoms was stronger among White compared to Black adolescents. The association between screen time and depressive symptoms was stronger among White compared to Asian adolescents.

**Conclusions:**

Screen time is prospectively associated with a range of mental health symptoms, especially depressive symptoms, though effect sizes are small. Video chat, texting, videos, and video games were the screen types with the greatest associations with depressive symptoms. Future research should examine potential mechanisms linking screen use with child behavior problems.

**Supplementary Information:**

The online version contains supplementary material available at 10.1186/s12889-024-20102-x.

## Introduction

Globally, mental disorders are significant contributors to disease burden and the leading cause of disability in adolescents (10–19 years) [1]. Research has documented the rising prevalence of adolescent mental health concerns in the United States. Adolescents are 50% more likely to experience a major depressive episode today than in the early 2000s [2]. Between 2000 and 2018, suicide rates increased by 30% in this population [3]. Internalizing (e.g., anxiety, depression) and externalizing (e.g., aggression, inattention) problems in childhood or adolescence have been linked to substance use and cognitive, psychosocial, and physical health impairments later in life [4–7]. Given that the peak and median age at onset for any mental disorder worldwide is 14.5 and 18 years, respectively [8], underlying factors contributing to the development of mental health problems during this developmental period may be important to target in interventions. Furthermore, the COVID-19 pandemic led to worse mental health among adolescents, with 42% of high school students reporting persistent feelings of sadness or hopelessness, a 50% increase from 2011 [9]. Despite the increasing prevalence and burden of mental health problems in adolescents, these factors are complex, intertwined, and poorly understood [1, 10].

An increase in the amount of time spent on screen-based technologies has been hypothesized to contribute to observed increases in the prevalence of mental health problems and suicide among adolescents [11–13]. Smartphones, tablets, television, and other screen-based technologies have become increasingly ubiquitous and embedded into family life [14]. On average, 8- to 12-year-olds spend 5.5 h per day using screen media, excluding time spent online for educational and homework purposes. For teenagers aged 13 to 18 years, screen time rises to 8.5 h per day [14]. Screen time in adolescents rose by 52% on average during the pandemic [15, 16]. Some research has demonstrated a link between self-reported screen time (total amount of time spent on screens; default measure of digital technology use in most studies to date) and poor mental health outcomes [17–20]. Increased screen time may be a possible reflection of problematic screen use, including difficulty self-regulating use and consequent personal, familial, social, and school-related functional impairments. Studies have linked increased screen exposure to decreased inhibitory control neurologically and behaviorally [21, 22]. Problematic screen use has been shown to be associated with poorer mental health in adolescents [23]. However, it should be noted that higher levels of screen exposure can represent both a cause and manifestation of behavioral and emotional symptoms [24].

This positive association between screen time and poorer mental health symptoms has prompted calls for guidelines to limit screen use among adolescents [25]. Some intervention studies, conducted primarily among adults, have shown that reductions in digital media use are associated with improvements in mental health outcomes, but other studies have also found no effect or negative consequences for well-being [26, 27]. A recent cluster randomized controlled trial found that adults who were allocated to reduce their household recreational digital screen use to less than three hours per week per person reported significantly improved mental well-being and mood at two-week follow-up [28]. Another randomized controlled trial found that reducing smartphone social media use in undergraduate students aged 16 to 24 years yielded significant improvements in appearance esteem and anxiety symptoms over four weeks [29].

However, the field has relied largely on cross-sectional and correlational data, with much of the conversation on screen time and mental health treating adolescents as a relatively uniform category without recognition of the potential differential impacts of screen time based on factors such as digital media modality, sex, and race/ethnicity [20]. Furthermore, a more detailed investigation of the associations between screen time and specific domains or even disorders of adolescent psychopathology is needed to provide more targeted recommendations and strategies.

The Child Behavior Checklist (CBCL), one of the most widely used and investigated tools for detecting emotional and behavioral symptoms in children and adolescents [30], provides a dimensional assessment of child psychopathology [31]. The CBCL includes *Diagnostic and Statistical Manual of Mental Disorders* (DSM)-oriented scales, which were developed based on expert consensus to be consistent with diagnostic categories from the DSM [32]. The DSM-oriented scales are as follows: affective/depressive, anxiety, attention-deficit/hyperactivity (ADHD), somatic, oppositional defiant (ODD), and conduct symptoms [31]. Studies have demonstrated an acceptable correspondence between the DSM-oriented scales and DSM diagnoses [33–40]. Although the scores in the clinical range for specific DSM-oriented scales of the CBCL are not directly equivalent to the corresponding specific diagnosis [41, 42], the CBCL’s DSM-oriented scales for depression, anxiety disorders, ADHD, somatic symptoms, ODD, and conduct disorders can be used in clinical settings for screening for psychopathology based on the DSM classification system and enhancing diagnostic assessment [40].

### Depression

Of the disorders included in the CBCL’s DSM-oriented scales, depression has been the most investigated in association with screen time. More screen time has been associated with depressive symptoms among children and adolescents in several systematic reviews [11, 12, 43–50]. In a systematic review of longitudinal studies examining the relationship between screen time and internalizing mental health symptoms, Tang et al. (2021) found a small but significant correlation between screen time and subsequent depressive symptoms among adolescents aged 10 to 24 years.

### Anxiety

In contrast to depressive symptoms, there are relatively few cross-sectional studies and even fewer longitudinal studies examining the association of screen time with anxiety, ADHD, somatic symptoms, ODD, and conduct disorders among children and adolescents [12, 51]. Some studies support a positive cross-sectional and longitudinal association between screen time and anxiety symptoms in adolescents [52, 53], but others found no significant association between screen time at baseline and changes in anxiety over time [54, 55]. Given the limited number of studies with mixed findings, systematic reviews have deemed the existing literature insufficient to draw conclusions [12, 45].

### Attention-deficit/hyperactivity disorder

Synthesizing data from eight cross-sectional and three longitudinal studies, a systematic review from 2015 concluded that there was strong evidence to support a positive association between screen time and hyperactivity/inattention symptoms in children and adolescents [56]. A more recent review evaluating the longitudinal associations between digital media use and ADHD symptoms found reciprocal associations between digital media use and ADHD symptoms [57].

### Somatic symptoms

Somatic symptom disorder is a psychiatric condition characterized by a significant focus on one or more physical symptoms, such as pain in different locations of the body, weakness, dizziness, nausea, and shortness of breath [58, 59]. Prior cross-sectional studies have examined the relationship between screen time and somatic symptoms in children, adolescents, and young adults [60–66], with the majority finding a positive association between screen time and somatic symptoms. To our knowledge, analyses of the longitudinal associations between screen time and somatic symptoms have not been published.

### Conduct disorder and oppositional defiant disorder

Similarly, previous cross-sectional studies have found potential associations between screen time and symptoms of conduct disorder and ODD among adolescents [67–72]. One study of 151 adolescents at risk for mental health symptoms found an association between average daily digital technology use and more conduct disorder symptoms both on the same day and 18 months later [73]. Consistent with these findings, our group has previously found higher screen time to be prospectively associated with higher odds of conduct disorder and ODD at one-year follow-up, based on longitudinal data from a larger (*n* = 11,875), national cohort of adolescents who participated in the ABCD Study [74].

### Gaps in prior literature

Certain methodological issues, such as sampling strategies and cross-sectional design, limit the generalizability of results across studies. For instance, few existing studies feature longitudinal time frames and account for additional demographic factors, particularly race/ethnicity and sex [12, 75, 76]. Accounting for potential moderators (e.g., sex and race/ethnicity) on the impact of screen exposure on adolescent mental health could help explain the heterogeneity seen across study findings. Additionally, investigating these potential moderators may improve the identification of at-risk populations and aid in the development of more targeted interventions [51, 76]. Prior studies have identified sex differences in the relationship between screen time and mental health outcomes, but this evidence remains inconsistent across studies [11, 12, 51], calling for additional longitudinal analyses to provide further insight into the moderating effect of sex. The moderating effect of race/ethnicity in the relationship between screen time and mental health has not been as extensively studied, although there are documented disparities in screen use [77–79] and mental health outcomes [80–83] across race/ethnicity in children and adolescents. For instance, data from the ABCD Study showed that, compared to White adolescents, Black adolescents reported greater total screen time use and Asian adolescents reported lower screen time use [77]. The same analysis found that, while male adolescents reported higher overall screen time than female adolescents, female adolescents reported higher daily use of social networking, texting, and video chatting [77]. Such differences by sex and race/ethnicity could be reflected in differences in associations between screen time and mental health outcomes which warrant further investigation.

Few studies examining longitudinal links between screen time and mental health symptoms have included large national cohorts of adolescents in North America. In a recent systematic review and meta-analysis on screen time and internalizing and externalizing behaviors among children and adolescents aged 12 years or younger [84], only three North American studies included a national cohort with a sample size of 10,000 or more [62, 85, 86]. Further, all three studies featured a cross-sectional study design and did not investigate the longitudinal relationship between screen time and internalizing and externalizing behaviors in adolescents. The cross-sectional design of the majority of these studies limits the ability to establish causal and temporal effects. Longitudinal studies provide more robust data and enable the examination of correlations over time [12].

Furthermore, it remains unclear whether specific modalities of screen time (e.g., device type, digital media type, and specific websites and applications) are differentially associated with adolescent mental health outcomes, prompting a call for researchers to conduct more nuanced measurements and analyses of screen use that focus on the contents, contexts, and environments in which digital media exposures occur [11, 51, 87–90]. To address such methodological limitations in existing studies, we aim to examine the longitudinal relationships between screen time (total aggregate screen time and specific types of screen time) and mental health symptoms measured by the CBCL’s DSM-oriented scales in a national cohort of adolescents in the United States [85]. Participants in the current analysis were 9 to 10 years old at baseline and were followed for two years. We hypothesized that higher screen time would be prospectively associated with higher scores on all CBCL DSM-oriented scales (anxiety, affective/depressive, somatic, ADHD, ODD, and conduct symptoms) at one- and two-year follow-up.

## Methods

### Study population

We used longitudinal data from baseline to Year 2 from the Adolescent Brain Cognitive Development (ABCD) Study (4.0 release). The ABCD Study is an ongoing prospective cohort study of health and cognitive development including 11,875 participants (ages 9–10 years at baseline in 2016–2018) from 21 recruitment sites across the U.S. The ABCD Study participants, recruitment, protocol, and measures are described in detail elsewhere [91]. Among 11,875 participants, 2,337 had missing data for total screen time and confounders, especially in Year 2, leaving 9,538 participants for the current analysis. Appendix A shows sociodemographic characteristics of participants who were included versus excluded from the current analysis. Institutional review board approval was received from the University of California, San Diego, and the respective IRBs of each study site. Written assent was obtained from participants, and written informed consent was obtained from their caregivers.

### Variables

#### Independent variable: screen time

Screen time was obtained from the ABCD Youth Screen Time Survey [92]. Participants were asked to answer questions about the number of hours per weekday/weekend day they spent on six different screen modalities (excluding school use), including watching/streaming TV shows or movies, watching/streaming videos [e.g., YouTube], playing videogames, texting, video chatting [e.g., Skype, Facetime], and social media [e.g., Facebook, Instagram, Twitter]. Total screen time was calculated separately for weekdays and weekend days, based on a previously validated measure [93–95]. The following formula was used to calculate the weighted average: [(weekday average x 5) + (weekend average x 2)/7] [62]. The weighted average of total screen time was reported as a continuous variable.

#### Dependent variables: Child Behavior Checklist (CBCL)

The CBCL is a screening tool consisting of 112 items asking a parent/caretaker about multiple behavioral, emotional, and mental health symptoms in children and adolescents aged 4 to 18 years [96, 97]. The CBCL included six DSM-oriented scales, including depressive, anxiety, somatic, attention-deficit/hyperactivity, oppositional defiant, and conduct symptoms. Parents/caretakers responded to statements about their child’s behavior using a scale from 0 (not true) to 2 (very true/often true) over the past six months. T-scores were calculated based on the CBCL scoring rubric. The CBCL has high test-retest reliability (ICC = 0.95), strong validity (ability of all items to discriminate significantly *p* < 0.01) [98], and acceptable internal consistency with alphas ranging from 0.63 to 0.79 [99]. Confirmatory factor analysis results for the DSM-oriented scales indicated good fit (Comparative Fit Index [CFI] of 0.96 and Root Mean Square Error of Approximation [RMSEA] of 0.045 [100, 101].

### Confounders

The following variables were used in statistical models as potential confounders of the association between baseline screen time and CBCL measures including age (years), sex (female, male), race/ethnicity (White, Latino/Hispanic, Black, Asian, Native American, and other), household income (U.S. dollars, six categories: less than $25,000, $25,000 through $49,999, $50,000 through $74,999, $75,000 through $99,999, $100,000 through $199,999, and $200,000 and greater), highest parent education (high school or less vs. college or more), and study site. Because the two-year follow-up data collection period (2018–2020) coincided with the COVID-19 pandemic, which affected both screen time and mental health, we controlled for the data collection period (before or during the COVID-19 pandemic, using March 13, 2020 as the start date of the COVID-19 pandemic in the US) in the analyses of the Year 2 data. In addition, sleep and physical activity could mediate the association between screen time and mental health, as more time on screens could displace time for sleep and physical activity, which are both beneficial for mental well-being. Sleep duration was measured by parent report based on an item from the Sleep Disturbance Scale for Children [102]. Physical activity was measured based on adolescent reports of the number of days in the last 7 days of spending at least 60 min per day physically active (the recommended daily level for children and adolescents from the Physical Activity Guidelines for Americans) [91, 103].

### Statistical analysis

We used total screen time and each of the six screen time components at baseline as the primary independent variable. The dependent variables were repeated measures from CBCL DSM-oriented scale scores derived as repeated measures of t-scores at each year, from baseline to Year 2. We used mixed-effects models with random effects to assess the association of baseline screen time with each CBCL DSM-oriented scale. Model 1 was unadjusted. In Model 2, the outcomes were CBCL DSM-oriented scale t-scores from Year 1 and Year 2, adjusted for baseline CBCL DSM-oriented scale t-scores and the following confounders at baseline: age, sex, race/ethnicity, household income, parent education, data collection period, and study site. We also conducted a supplemental analysis adjusting for sleep and physical activity in addition to age, sex, race/ethnicity, household income, parent education, data collection period, and study site. We tested for effect modification by sex and race/ethnicity in the association between screen time and CBCL DSM-oriented scales. We present results stratified by sex or race/ethnicity for behavioral outcomes where there was evidence of effect modification by sex or race/ethnicity, respectively (*p* for interaction < 0.05). *P*-values < 0.05 were considered to indicate statistical significance. Data analyses were performed using Stata 18.0 (College Station, TX) and applied propensity weights based on the American Community Survey [104].

## Results

Characteristics of the 9,538 participants are shown in Table 1. The mean age at baseline was 9.9 ± 0.6 years; 51.2% of the participants were male, and 47.6% were non-White. The average total screen time at baseline was 4.0 ± 3.2 h per day, with most time spent watching television shows/movies (1.3 ± 1.1 h/day), watching/streaming videos (1.3 ± 1.2 h/day) and playing video games (1.2 ± 1.1 h/day). Furthermore, somatic symptoms had the highest t-score (55.4), among the CBCL DSM-oriented scales (Table 1).

**Table 1:** Sociodemographic, screen time, and mental health characteristics of 9,538 Adolescent Brain Cognitive Development (ABCD) Study participants at baseline (2016–2018)

Sociodemographic characteristics	Mean (SD) / %
Age (years), mean (SD)	9.9 (0.6)
Sex (%)	
Female	48.8%
Male	51.2%
Race/ethnicity (%)	
White	52.4%
Latino / Hispanic	20.1%
Black	17.3%
Asian	5.5%
Native American	3.2%
Other	1.5%
Household income (%)	
Less than $25,000	18.1%
$25,000 through $49,999	20.7%
$50,000 through $74,999	18.0%
$75,000 through $99,999	15.6%
$100,000 through $199,999	20.9%
$200,000 and greater	6.7%
Parent with college education or more (%)	79.7%
**Recreational screen time variables**	
Total screen time, hours per day, mean (SD)	4.0 (3.2)
Television shows/movies, hours per day, mean (SD)	1.3 (1.1)
Videos (e.g. YouTube), hours per day, mean (SD)	1.3 (1.2)
Video games, hours per day, mean (SD)	1.2 (1.1)
Texting, hours per day, mean (SD)	0.2 (0.6)
Video chat, hours per day, mean (SD)	0.3 (0.7)
Social media, hours per day, mean (SD)	0.1 (0.1)
**Mental health symptoms (Child Behavior Checklist t-score)**	
Depressive symptoms	53.9 (6.1)
Anxiety symptoms	53.6 (6.3)
Somatic symptoms	55.4 (6.6)
Attention-deficit/hyperactivity symptoms	53.2 (5.6)
Oppositional defiant symptoms	53.4 (5.4)
Conduct symptoms	52.9 (5.4)

Propensity weights were applied to yield representative estimates based on the American Community Survey from the US Census. SD = standard deviation

Table 2 shows the unadjusted (Model 1) and adjusted (Model 2) models for associations between total screen time and CBCL DSM-oriented symptom scale t-scores. Higher total screen time was associated with all DSM-oriented scales in adjusted models (Model 2), and the association was strongest for depressive symptoms (B = 0.10, 95% CI 0.06, 0.13, *p* < 0.001), conduct symptoms (B = 0.07, 95% CI 0.03, 0.10, *p* < 0.001), somatic symptoms (B = 0.06, 95% CI 0.01, 0.11, *p* = 0.026), and attention-deficit/hyperactivity symptoms (B = 0.06, 95% CI 0.01, 0.10, *p* = 0.013). Supplemental analyses adjusting for sleep and physical activity in addition to the covariates adjusted for in Model 2 showed similar results although some associations were slightly attenuated (Appendix B).

**Table 2:** Prospective associations between screen time and its subtypes with mental health symptoms in the Adolescent Brain Cognitive Development (ABCD) Study

	Depressive symptoms		Anxiety symptoms		Somatic symptoms		Attention-deficit/hyperactivity symptoms		Oppositional defiant symptoms		Conduct symptoms	
**Model 1: Unadjusted**	Coefficient (95% CI)	*p*	Coefficient (95% CI)	*p*	Coefficient (95% CI)	*p*	Coefficient (95% CI)	*p*	Coefficient (95% CI)	*p*	Coefficient (95% CI)	*p*
Total screen time	**0.18 (0.14**,** 0.22)**	**< 0.001**	**0.12 (0.06**,** 0.17)**	**< 0.001**	**0.10 (0.05**,** 0.14)**	**< 0.001**	**0.27 (0.22**,** 0.31)**	**< 0.001**	**0.21 (0.15**,** 0.27)**	**< 0.001**	**0.28 (0.21**,** 0.36)**	**< 0.001**
Television shows/movies	**0.32 (0.15**,** 0.48)**	**0.001**	0.19 (-0.02, 0.39)	0.428	0.17 (-0.003, 0.36)	0.055	**0.50 (0.32**,** 0.69)**	**< 0.001**	**0.43 (0.27**,** 0.57)**	**< 0.001**	**0.57 (0.32**,** 0.82)**	**< 0.001**
Videos (e.g. YouTube)	**0.50 (0.37**,** 0.64)**	**< 0.001**	**0.36 (0.22**,** 0.51)**	**< 0.001**	**0.34 (0.22**,** 0.47)**	**< 0.001**	**0.56 (0.46**,** 0.65)**	**< 0.001**	**0.42 (0.29**,** 0.55)**	**< 0.001**	**0.52 (0.41**,** 0.65)**	**< 0.001**
Video games	**0.48 (0.37**,** 0.60)**	**< 0.001**	**0.30 (0.16**,** 0.44)**	**< 0.001**	**0.19 (0.07**,** 0.32)**	**0.003**	**0.61 (0.50**,** 0.72)**	**< 0.001**	**0.48 (0.32**,** 0.63)**	**< 0.001**	**0.53 (0.38**,** 0.68)**	**< 0.001**
Texting	**0.22 (0.06**,** 0.37)**	**0.008**	0.04 (-0.15, 0.22)	0.690	0.18 (-0.19, 0.54)	0.331	**0.46 (0.27**,** 0.65)**	**< 0.001**	**0.38 (0.11**,** 0.66)**	**< 0.001**	**0.75 (0.37**,** 1.13)**	**< 0.001**
Video chat	**0.29 (0.07**,** 0.51)**	**0.012**	0.02 (-0.22, 0.28)	0.536	0.07 (-0.21, 0.36)	0.589	**0.51 (0.29**,** 0.74)**	**< 0.001**	**0.43 (0.07**,** 0.80)**	**0.023**	**0.80 (0.43**,** 1.17)**	**< 0.001**
Social media	**0.44 (0.24**,** 0.64)**	**< 0.001**	0.08 (-0.19, 0.37)	0.536	0.28 (-0.04, 0.60)	0.078	**0.68 (0.40**,** 0.96)**	**< 0.001**	**0.71 (0.44**,** 0.98)**	**< 0.001**	**1.15 (0.86**,** 1.46)**	**< 0.001**
**Model 2: Adjusted for sociodemographic factors and baseline mental health**												
Total screen time	**0.10 (0.06**,** 0.13)**	**< 0.001**	**0.05 (0.01**,** 0.09)**	**0.029**	**0.06 (0.01**,** 0.11)**	**0.026**	**0.06 (0.01**,** 0.10)**	**0.013**	**0.04 (0.01**,** 0.07)**	**0.011**	**0.07 (0.03**,** 0.10)**	**< 0.001**
Television shows/movies	**0.13 (0.01**,** 0.26)**	**0.036**	0.06 (-0.09, 0.21)	0.397	0.04 (-0.09, 0.16)	0.559	0.11 (-0.01, 0.24)	0.067	**0.10 (0.01**,** 0.19)**	**0.032**	0.11 (-0.01, 0.22)	0.063
Videos (e.g. YouTube)	**0.22 (0.13**,** 0.31)**	**< 0.001**	**0.17 (0.08**,** 0.25)**	**0.001**	**0.19 (0.09**,** 0.29)**	**0.001**	**0.09 (0.004**,** 0.17)**	**0.042**	0.07 (-0.02, 0.15)	0.114	**0.10 (0.02**,** 0.18)**	**0.018**
Video games	**0.20 (0.03**,** 0.37)**	**0.022**	0.08 (-0.02, 0.17)	0.099	0.13 (-0.001, 0.27)	0.05	**0.11 (0.05**,** 0.18)**	**0.001**	0.07 (-0.01, 0.15)	0.07	**0.10 (0.05**,** 0.16)**	**0.001**
Texting	**0.26 (0.09**,** 0.44)**	**0.005**	0.10 (-0.07, 0.27)	0.231	0.19 (-0.04, 0.42)	0.101	0.18 (-0.02, 0.39)	0.080	0.12 (-0.04, 0.28)	0.151	**0.33 (0.11**,** 0.56)**	**0.006**
Video chat	**0.35 (0.19**,** 0.51)**	**< 0.001**	0.12 (-0.06, 0.30)	0.189	0.05 (-0.21, 0.31)	0.683	**0.22 (0.03**,** 0.41)**	**0.022**	0.11 (-0.09, 0.33)	0.246	**0.35 (0.14**,** 0.57)**	**0.002**
Social media	0.14 (-0.09, 0.33)	0.230	0.004 (-0.24, 0.25)	0.971	0.11 (-0.16, 0.38)	0.421	0.04 (-0.31, 0.39)	0.805	0.07 (-0.19, 0.33)	0.617	0.22 (-0.02, 0.47)	0.071

Models represent the abbreviated outputs from mixed effects models examining associations between screen time and its subtypes (independent variable at baseline) and mental health symptoms (dependent variable at one- and two-year follow-up based on the Child Behavior Checklist [CBCL]). Propensity weights from the ABCD Study were applied based on the American Community Survey from the US Census

Model 1 is unadjusted

Model 2 includes random effects adjusted for age, race/ethnicity, household income, parent education, study site, baseline CBCL score, and date of CBCL administration

We stratified results by race/ethnicity for outcomes where there was evidence of significant effect modification by race/ethnicity on the associations between total screen time and CBCL DSM-oriented symptom scales. In adjusted models (Table 3), screen time was associated with higher depressive (B = 0.13, 95% CI 0.09, 0.17), attention-deficit/hyperactivity (B = 0.07, 95% CI 0.02, 0.13), and oppositional defiant (B = 0.05, 95% CI 0.01, 0.10) symptom scores in White adolescents but not among Black adolescents. The association between screen time and depressive symptoms was stronger among White compared to Asian adolescents. There was no evidence of effect modification of screen time by sex for any of the outcomes (*p* for screen time*sex interaction > 0.05).

**Table 3:** Prospective associations between total screen time and mental health symptoms in the Adolescent Brain Cognitive Development (ABCD) Study, stratified by race/ethnicity

	Stratified by race/ethnicity										
	White subsample		Black subsample			Asian subsample			Native American subsample		
	Coefficient (95% CI)	*p*	Coefficient (95% CI)	*p*	*p* ^a^	Coefficient (95% CI)	*p*	*p* ^a^	Coefficient (95% CI)	*p*	*p* ^a^
Depressive symptoms	0.13 (0.09, 0.17)	< 0.001	0.02 (-0.04, 0.09)	0.387	0.003	0.02 (-0.08, 0.13)	0.882	0.034	--	--	--
Anxiety symptoms	--	--	--	--	--	--	--	--	**--**	--	--
Somatic symptoms	0.10 (0.04, 0.16)	0.001	--	--	--	--	--	--	-0.17 (0.32, -0.01)	0.036	0.003
Attention-deficit/hyperactivity symptoms	0.07 (0.02, 0.13)	0.015	0.01 (-0.04, 0.06)	0.604	0.017	--	--	--	--	--	--
Oppositional defiant symptoms	0.05 (0.01, 0.10)	0.024	-0.02 (-0.07, 0.03)	0.395	0.019	--	--	--	--	--	--
Conduct symptoms	--	--	--	--	**--**	--	--	--	--	--	--

Models represent the abbreviated outputs from mixed effects models examining associations between screen time (independent variable at baseline) and mental health symptoms (dependent variable at one- and two-year follow-up based on the Child Behavior Checklist [CBCL]). Models include random effects adjusted for age, household income, parent education, study site, baseline CBCL score, and date of CBCL administration. Propensity weights from the ABCD Study were applied based on the American Community Survey from the US Census. Results stratified by race/ethnicity are only presented for mental health symptoms where there was evidence of effect modification by race/ethnicity

^a^*P*-value for the screen time*race/ethnicity interaction term coefficient

## Discussion

In a demographically diverse, nationwide, longitudinal cohort of 9,538 early adolescents in the United States, the current study found that higher total screen time was prospectively associated with higher scores on all DSM-oriented scales of the CBCL at both one- and two-year follow-up, even after adjusting for confounders. These results were held after adjusting for CBCL DSM-oriented scores at baseline. The specific DSM-oriented scale most strongly associated with total screen time was depressive symptoms. In this study, the average total screen time at baseline, when participants were 9 to 10 years old, was 4.0 ± 3.2 h per day. While the digital and in-person socialization landscape during the study’s baseline period (2016 to 2018) is distinct from that of the contemporary context, the average total screen time of this study’s sample is comparable to more recent national statistics for average screen time among children and younger adolescents aged 8 to 12 years in 2021 (5.5 h per day) [14].

The present study adds to the current literature on the relationship between screen time and adolescent mental health by assessing the longitudinal impact of different screen time modalities on specific domains of adolescent psychopathology that have clinical relevance. Recent reviews and meta-analyses have concluded that the literature on the mental health impacts of screen time among adolescents presents mixed findings that are difficult to collectively interpret [75, 105, 106], highlighting the need to consider different modalities of screen time [11, 51, 87, 89, 90], control for demographic variables and other potential confounders [107], and include more longitudinal perspectives [75, 108, 109].

Consistent with previous analyses, which have included longitudinal data and larger cohorts other than the ABCD Study cohort, we found weak but significant correlations between screen time and adolescents’ internalizing and externalizing behavior symptoms, including depression, anxiety, ADHD, somatic, ODD, and conduct symptoms [12, 57, 84, 110, 111]. There are various factors to consider when interpreting the small effect sizes. While some have suggested that the small effect sizes suggest a small or even negligible impact of increased screen time on the prevalence of mental health symptoms among adolescents [12], others have suggested that the consequences of screen time at a population level are likely meaningful despite small effect sizes [84, 112]. Regarding the interpretation of longitudinal effect sizes, it has been argued that even small associations may be of importance when controlling for baseline levels [113]. Controlling for stability effects often attenuates the magnitude of effect size coefficients in longitudinal designs. It is thus misleading to apply the same guidelines for interpreting longitudinal effect size coefficients in models that control for stability effects versus cross-sectional effect size coefficients in analyses that control for confounds, but not stability effects [113]. Further, the effect sizes reported are for each hour of screen time; given that average screen time for adolescents rose to nearly eight hours per day during the COVID-19 pandemic, these effects could be magnified [16]. These effect sizes per hour of screen time are similar in magnitude to the effect sizes previously reported on screen time and nutrition as measured by the MIND (Mediterranean-DASH [Dietary Approaches to Stop Hypertension] Intervention for Neurodegenerative Delay) diet score [114].

In this study, the specific DSM-oriented scale most strongly associated with screen time was depressive symptoms. These findings may be explained, in part, by some combination of various media effects theories that have been proposed [115], including the displacement hypothesis [116, 117]. The displacement hypothesis posits that screen time may replace time adolescents spend engaging in physical activity, sleep, in-person interactions, and other beneficial pursuits demonstrated to help reduce depression and anxiety symptoms [118–120]. Studies have also shown that higher levels of screen time were associated with reduced sleep duration and more sleep disturbances, which were in turn associated with internalizing, externalizing, and peer problems [62, 121]. The weaker but still significant associations between screen time and depressive symptoms, along with the other assessed CBCL DSM-oriented scales found after adjusting for sleep and physical activity (i.e., displacement hypothesis) in Appendix B suggest that displacement theory partially accounts for, but does not fully explain, the relationship between screen time and early adolescents’ mental health symptoms.

The specific screen types with the greatest associations with depression include video chat, texting, videos (e.g., YouTube), and video games. Of note, there was not a statistically significant association between social media and depression or any of the mental health outcomes, although the coefficients were all in the positive direction. This may be due to the fact that participants’ age during the data collection period for social media screen time (9–10 years old) is younger than the minimum age requirement to have a social media account (13 years old). Thus, participants on average reported spending the least screen time on social media, out of all the screen types assessed.

### Moderating effect of race/ethnicity in the prospective relationship between screen time and mental health

The present study investigated the impact of race/ethnicity as a moderator in the association between screen time and mental health symptoms, demonstrating a significant association between total screen time and depressive, ADHD, and ODD symptoms in White adolescents, but not in Black adolescents. This suggests that the longitudinal associations between screen time and several mental health symptoms are significantly weaker among Black adolescents than White adolescents. In addition, the association between total screen time and depressive symptoms was stronger among White compared to Asian adolescents. The extant literature on the impact of screen exposure on the psychosocial outcomes of racial and ethnic minority adolescents in the United States is sparse [122–124]. However, it is possible that adolescents from racial/ethnic minority backgrounds who might experience isolation, bullying, or discrimination in person may use screens to connect with others with similar backgrounds, which could buffer from depression, anxiety, and other symptoms of poor mental health [125]. Further research is needed to further elucidate potential differences by race/ethnicity. Other possible explanations include cultural variability in symptom presentation, which may not be comprehensively captured by the diagnostic classification system [126]. Furthermore, as parents complete rating scales in the CBCL, they may make implicit comparisons to a culturally-based standard for how children should behave or to their child’s local peers [127]. Internalized stigma about mental health may dissuade individuals from reporting symptoms or seeking help and services [126].

### Strengths and limitations

Strengths of this study include the longitudinal data spanning two years of follow-up in a large, nationwide sample of adolescents in the US that was diverse, allowing the examination of moderation of effects by sex and race/ethnicity between screen time and mental health symptoms. Limitations should also be noted. Screen time was based on self-report which could be subject to response, recall, and social desirability bias. Screen time does not capture the content or context of screen use, which could be examined in future research [20, 128]. The current analysis was limited by the availability of data from the ongoing ABCD Study and could only follow adolescents for two years, starting from age 9 to 10. However, given that digital technology use among children increases with age, particularly during adolescence [129, 130], it is important to continue characterizing the relationships between digital technology use and mental health over time. Although we examined the prospective association of screen time leading to mental health outcomes, there is the possibility of inverse causality. Bidirectional associations between screen time and mental health could be supported by the self-perpetuating feedback loop model [131], whereby screen use leads to worsening mental health and poor mental health leads to increasing reliance on screens to cope [132]. Although we controlled for age, sex, race/ethnicity, household income, parent education, and study site, there is the possibility of unmeasured confounders. The effect sizes were relatively small.

## Conclusion

Our longitudinal study identified several important prospective associations between screen time and DSM-oriented symptoms in a national sample of adolescents, most notably depression and conduct symptoms. These findings can help to inform developmentally appropriate guidance related to screen use, especially for adolescents and their parents. The American Academy of Pediatrics advocates for a Family Media Use plan for children 5 to 18 years old [133], which could be individualized for adolescents based on some of the associations noted in the current study, and nuances in some associations by sex and race/ethnicity. Education, prevention, and intervention efforts may be particularly important in early adolescence given that depression and other mental health conditions increase in mid- to late-adolescence; therefore, acting of modifiable behaviors in early adolescence could be protective. Future research could examine longer-term associations with additional years of follow-up as the ABCD Study cohort ages through mid-to-late adolescence.

## Electronic supplementary material

Below is the link to the electronic supplementary material.

Supplementary Material 1

## Data Availability

Data used in the preparation of this article were obtained from the ABCD Study (https://abcdstudy.org), held in the NIMH Data Archive (NDA). Investigators can apply for data access through the NDA (https://nda.nih.gov/).

## Abbreviations

ABCD: Adolescent Brain Cognitive Development Study
ADHD: Attention-deficit/hyperactivity
CBCL: Child Behavior Checklist
CFI: Comparative Fit Index
DSM: Diagnostic and Statistical Manual of Mental Disorders
IRB: Institutional review board
ODD: Oppositional defiant
RMSEA: Root Mean Square Error of Approximation
