# Supplementary material for: Screen time and mental health: a prospective analysis of the Adolescent Brain Cognitive Development (ABCD) Study
Source: BMC Public Health. 2024 Oct 7;24:2686. doi: 10.1186/s12889-024-20102-x (PMC11457456; doi:10.1186/s12889-024-20102-x)
Supplement: Supplementary file 1 — Supplementary Material 1 [file 12889_2024_20102_MOESM1_ESM.docx]

| Appendix A: Comparison in baseline characteristics between participants with and without missing data | | | |
| --- | --- | --- | --- |
|  | Participants without missing data n=9,538 | Participants with missing data n=2,337 |  |
| Sociodemographic characteristics (baseline) | Mean (SD) / % | Mean (SD) / % | p |
| Age (years), mean (SD) | 9.9 (0.6) | 9.9 (0.6) | 0.776 |
| Sex (%) |  |  |  |
| Female | 48.80% | 49.20% | 0.832 |
| Male | 51.20% | 50.80% |  |
| Race/ethnicity (%) |  |  |  |
| White | 52.40% | 38.30% | <0.001 |
| Latino / Hispanic | 20.10% | 24.40% |  |
| Black | 17.30% | 26.95% |  |
| Asian | 5.50% | 5.60% |  |
| Native American | 3.20% | 3.00% |  |
| Other | 1.50% | 1.75% |  |
| Household income (%) |  |  |  |
| Less than $25,000 | 18.10% | 30.60% | <0.001 |
| $25,000 through $49,999 | 20.70% | 23.50% |  |
| $50,000 through $74,999 | 18.00% | 16.65% |  |
| $75,000 through $99,999 | 15.60% | 10.90% |  |
| $100,000 through $199,999 | 20.90% | 13.90% |  |
| $200,000 and greater | 6.70% | 4.45% |  |
| Parent with college education or more (%) | 79.70% | 66.80% | <0.001 |

| Appendix B. Prospective associations between screen time and its subtypes with mental health symptoms, adjusting for sleep and physical activity, in the Adolescent Brain Cognitive Development (ABCD) Study | | | | | | | | | | | | |
| --- | --- | --- | --- | --- | --- | --- | --- | --- | --- | --- | --- | --- |
|  | Depressive symptoms | | Anxiety symptoms | | Somatic symptoms | | Attention-deficit/hyperactivity symptoms | | Oppositional defiant symptoms | | Conduct symptoms | |
|  | Coefficient (95% CI) | p | Coefficient (95% CI) | p | Coefficient (95% CI) | p | Coefficient (95% CI) | p | Coefficient (95% CI) | p | Coefficient (95% CI) | p |
| Total screen time | **0.09 (0.06, 0.12)** | **<0.001** | **0.05 (0.0004, 0.09)** | **0.048** | **0.06 (0.004, 0.11)** | **0.037** | **0.05 (0.01, 0.10)** | **0.019** | **0.04 (0.01, 0.07)** | **0.013** | **0.08 (0.03, 0.10)** | **<0.001** |
| Television shows/movies | **0.13 (0.002, 0.26)** | **0.047** | 0.06 (-0.09, 0.21) | 0.428 | 0.03 (-0.10, 0.16) | 0.639 | 0.11 (-0.02, 0.24) | 0.081 | **0.10 (0.01, 0.20)** | **0.033** | 0.10 (-0.01, 0.23) | 0.077 |
| Videos (e.g. YouTube) | **0.21 (0.12, 0.30)** | **<0.001** | **0.16 (0.06, 0.25)** | **0.002** | **0.17 (0.07, 0.27)** | **0.002** | 0.08 (-0.01, 0.17) | 0.065 | 0.07 (-0.02, 0.15) | 0.123 | **0.09 (0.01, 0.18)** | **0.033** |
| Video games | **0.16 (0.10, 0.22)** | **<0.001** | 0.07 (-0.02, 0.16) | 0.122 | 0.13 (-0.01, 0.26) | 0.066 | **0.11 (0.05, 0.18)** | **0.002** | 0.08 (-0.01, 0.16) | 0.069 | **0.10 (0.04, 0.16)** | **0.002** |
| Texting | **0.25 (0.09, 0.42)** | **0.004** | 0.10 (-0.07, 0.28) | 0.234 | 0.18 (-0.05, 0.42) | 0.117 | 0.18 (-0.03, 0.38) | 0.085 | 0.12 (-0.05, 0.29) | 0.155 | **0.33 (0.10, 0.56)** | **0.007** |
| Video chat | **0.35 (0.18, 0.51)** | **<0.001** | 0.12 (-0.06, 0.31) | 0.190 | 0.05 (-0.21, 0.30) | 0.708 | **0.22 (0.03, 0.40)** | **0.023** | 0.12 (-0.09, 0.33) | 0.249 | **0.35 (0.14, 0.56)** | **0.003** |
| Social media | 0.13 (-0.09, 0.37) | 0.246 | 0.001 (-0.25, 0.26) | 0.992 | 0.09 (-0.17, 0.36) | 0.484 | 0.03 (-0.31, 0.38) | 0.844 | 0.06 (-0.19, 0.32) | 0.621 | 0.22 (-0.03, 0.46) | 0.081 |
| Models represent the abbreviated outputs from mixed effects models examining associations between screen time and its subtypes (independent variable at baseline) and mental health symptoms (dependent variable at one- and two-year follow-up based on the Child Behavior Checklist [CBCL]). Propensity weights from the ABCD Study were applied based on the American Community Survey from the US Census. | | | | | | | | | | | | |
| Model includes random effects adjusted for age, race/ethnicity, household income, parent education, study site, baseline CBCL score, date of CBCL administration, physical activity, and sleep problems. | | | | | | | | | | | | |
